# Role of Environmental Chemistry in Governing the Corrosion and Stress Corrosion Cracking Mechanism of L415 Pipeline Steel in Acidic Soils

**DOI:** 10.3390/ma18245492

**Published:** 2025-12-06

**Authors:** Siwen Liu, Minghao Liu, Yangqin Shangguan, Ke Mei, Shiyao Zhu, Kai Liu, Ruiquan Liao

**Affiliations:** 1 State Key Laboratory of Low Carbon Catalysis and Carbon Dioxide Utilization, Yangtze University, Wuhan 430100, China; 2 Hubei Key Laboratory of Oil and Gas Drilling and Production Engineering, Yangtze University, Wuhan 430100, China19871235649@163.com (S.Z.); 3 School of Petroleum Engineering, Yangtze University, Wuhan 430100, China

**Keywords:** L415 pipeline steel, acidic soil, acid corrosion, stress corrosion cracking, microstructure

## Abstract

The operational integrity of L415 pipeline steel, a critical component of China’s energy network, is severely threatened by the unique acidic red soil environments prevalent in Southern China. A significant knowledge gap exists regarding its specific failure mechanisms, particularly the interplay between Anodic Dissolution (AD) and Hydrogen Embrittlement (HE) in driving Stress Corrosion Cracking (SCC). This study systematically investigates the corrosion and SCC behavior of L415 steel in a simulated environment that replicates the typical soil chemistry of the Gannan region in Southern China. Results revealed that corrosion kinetics are highly dependent on environmental chemistry, with corrosion rates escalating nearly four-fold from 0.0505 mm/a to a severe 0.1949 mm/a, driven by the synergy of low pH and high SO_4_^2−^ concentration. This behavior is governed by the integrity of the corrosion product film, where aggressive environments form porous, unprotective layers with low charge transfer resistance. Slow strain rate tensile (SSRT) tests confirmed that the steel’s susceptibility to SCC is strongly promoted by acidity. Critically, the dominant SCC mechanism was environment-dependent, transitioning from Hydrogen Embrittlement (HE) to intergranular cracking in the most acidic environment, and a mixed AD-HE mechanism causing transgranular cracking in high-chloride conditions. These findings provide a direct mechanistic link between soil chemistry and failure mode, offering a crucial scientific basis for developing environment-specific integrity management strategies for pipelines in these challenging terrains.

## 1. Introduction

With the rapid expansion of China’s oil and gas pipeline network, ensuring its operational integrity has become a cornerstone of the national energy strategy [1,2]. These buried steel pipelines face a multitude of complex environmental challenges, with corrosive soil being one of the main causes of pipeline failure [3,4]. The accelerating effect of environmental factors can be dramatic; for instance, research has demonstrated that in a simulated soil environment, external stray currents can cause the corrosion rate of X52 steel to escalate from a baseline of 0.19 mm/a to 92.48 mm/a [5]. This dramatic effect from a single variable illustrates the critical importance of identifying and quantifying every key environmental factor that could potentially accelerate pipeline degradation [6,7].

A particularly challenging scenario is presented by the vast expanses of acidic red soils prevalent throughout Southern China, where a large number of pipelines are operated in environments with an average soil pH of 3.5–6.0 [8,9,10]. The high corrosivity of such acidic conditions is widely acknowledged and is potent enough to compromise even highly resistant materials; studies have shown that while AISI 304 stainless steel is inert in neutral pH soils, it suffers from pitting corrosion when the soil is acidified [11]. Long-term field burial tests have confirmed the persistent threat in this region, revealing that X70 steel buried in Southern China’s red soil exhibits an initial corrosion rate of 0.053 mm/year, with damage manifesting as a dangerous combination of non-uniform and pitting corrosion [12]. Furthermore, corrosion behavior is highly dependent on the specific steel grade and microstructure: a fact highlighted by research showing significant differences in corrosion rates between X60, X65, and X70 steels in the same acidic soil Among these, X60 demonstrates higher corrosion susceptibility in soil compared to X65 and X70 steels [13], while L415 steel exhibits comparable performance to X60 due to their similar metallurgical characteristics. This material-specificity underscores the imperative to conduct targeted research on the materials actually in service. In response to this need, this study focuses on L415 grade steel, which is a material widely used in China’s pipeline network, aiming to fill the critical data gap regarding its specific corrosion behavior in this uniquely challenging acidic soil environment.

Beyond accelerated material loss, the integrity of pipelines in these aggressive environments is threatened by a more insidious failure mode: Stress Corrosion Cracking (SCC) [14,15]. Unlike predictable uniform corrosion, SCC can lead to catastrophic brittle fracture of the pipeline with little or no macroscopic deformation, posing a more severe threat to operational safety [16]. This phenomenon occurs through the synergistic interaction of a susceptible material, a specific corrosive environment, and a sustained tensile stress [17], which is a condition inherent in pressurized pipelines. The risk is further amplified by local factors such as coating defects, which create “large cathode–small anode” galvanic cells that dramatically increase local current density, leading to rapid pitting that serves as preferential sites for SCC initiation [18].The mechanism of SCC in acidic soils is particularly complex and is governed by a competitive interplay between Anodic Dissolution (AD) and Hydrogen Embrittlement (HE) [19]. In the AD mechanism, stress concentration at a crack tip locally accelerates the dissolution of metal, leading to crack initiation and propagation. Concurrently, the cathodic reaction in the acidic environment produces a high concentration of hydrogen atoms. These atoms can permeate the steel lattice and accumulate at microstructural features like dislocations and inclusions, severely reducing the material’s toughness and inducing cracking via the HE mechanism. Critically, these two pathways are not mutually exclusive; the dominant mechanism is highly dependent on specific electrochemical conditions, environmental pH, and ionic composition, representing a development in the understanding of pipeline failure [20]. Despite this mechanistic understanding, a critical research gap exists at the intersection of material, environment, and failure mode. For widely deployed L415 pipeline steel, there is a lack of systematic studies connecting the specific chemistry of Southern China’s acidic soils (e.g., pH, Cl^−^, SO_4_^2−^) to its corrosion kinetics, protective film properties, and, most importantly, its SCC susceptibility. The dominant failure mechanism under the coupled influence of tensile stress and this soil chemistry—whether it is governed by Anodic Dissolution or Hydrogen Embrittlement—remains an open and critical question.

Therefore, this study aims to bridge this gap by systematically investigating the corrosion and SCC behavior of L415 steel in a simulated environment replicating the typical acidic soil chemistry of the Gannan region, a representative area of Southern China’s expansive red soil belt. By integrating weight-loss tests, electrochemical measurements, microstructural characterization, and slow strain rate tensile (SSRT) tests, we elucidate the influence of key environmental factors on corrosion kinetics and SCC susceptibility. The objective is to establish a comprehensive understanding of the degradation mechanisms, thereby providing a scientific basis for the integrity management and corrosion protection strategies for pipelines operating in this challenging environment.

## 2. Experimental Materials and Methods

### 2.1. Experimental Material and Specimen Preparation

**Figure 1:**
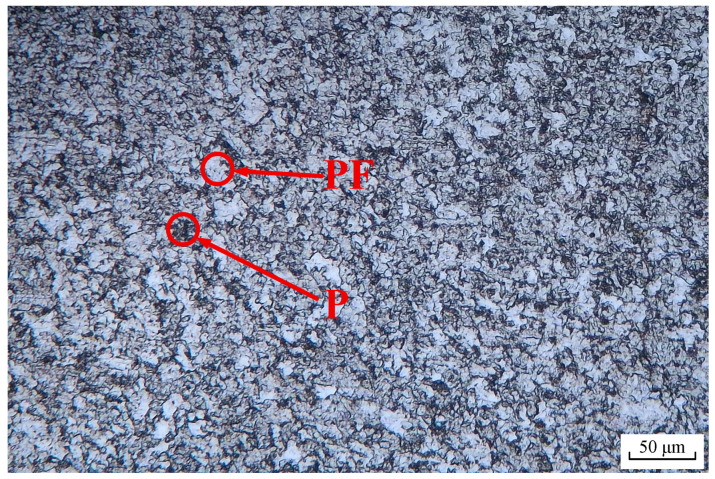
Microstructure of L415 pipeline steel.

The material used in this study was L415 pipeline steel (Baowu Steel Group, Shanghai, China), compliant with the API 5L specification [21]. Its chemical composition (wt.%) is detailed in Table 1. The microstructure, shown in Figure 1, was examined by optical microscopy after standard metallographic preparation and etching with 4% Nital solution. It consists of irregular ferrite (PF) and pearlite (P) phases.

**Figure 2:**
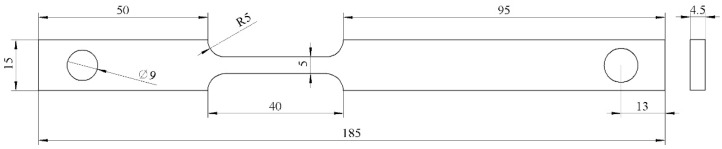
Slow Strain Rate Tensile (SSRT) specimens.

Three types of specimens were machined from the L415 steel plate:

1. Weight-loss specimens: Rectangular coupons with dimensions of 40 mm × 13 mm × 2 mm.
2. Electrochemical specimens: 10 mm × 10 mm × 3 mm blocks, with a copper wire welded to the back and mounted in epoxy resin, leaving an exposed working area of 1.0 cm^2^.
3. Slow Strain Rate Tensile (SSRT) specimens (shown in Figure 2): Dog-bone shaped tensile specimens with a gauge section of 185 mm × 15 mm × 4.5 mm. The tensile axis was parallel to the pipe rolling direction.

All working surfaces were mechanically ground with silicon carbide (SiC) papers down to 1200 grit. After grinding, specimens were ultrasonically cleaned in deionized water and ethanol, then dried in cool air. Prior to experiments, all specimens were sterilized under a UV lamp (Xinyate, Suzhou, China, T8) for 30 min to prevent microbial influence.

### 2.2. Soil Sampling and Physicochemical Analysis

To create a corrosive environment representative of field conditions, four typical acidic soils were systematically collected along an in-service oil and gas pipeline in the Gannan region of Southern China. Soil samples were taken at a depth of approximately 1.5 m, corresponding to the pipeline’s burial depth. In the laboratory, the samples were air-dried, and impurities such as plant roots were removed. Subsequently, soil was ground and passed through a 20-mesh sieve before being sealed for storage.

To quantitatively assess the corrosivity of the soil samples, soil leachates were prepared using a soil-to-water mass ratio of 1:5, following standard (LY/T 1251-1999) methods [22]. The key physicochemical properties were analyzed. The pH value was measured with a precision pH meter (Leici, Shanghai, China, PHSJ-3F). The concentrations of major anions (Cl^−^, SO_4_^2−^, HCO_3_^−^) were determined by ion chromatography (SHINE, Qingdao, China, CIC-D100). The total dissolved solids (TDS) were calculated by summing the concentrations of these ions. The detailed physicochemical parameters of the soil samples are summarized in Table 2. The results revealed distinct differences among the environments. Soil A129 was the most acidic, with a pH value as low as 3.40. Soil B041 was characterized by an extremely high concentration of SO_4_^2−^ (244.57 mg/L), leading to the highest Total Dissolved Solids (TDS) value of 506.18 mg/L, indicating the highest conductivity. Soil A107 exhibited a significantly higher Cl^−^ concentration (29.66 mg/L) compared to the others. In contrast, soil B080 was the mildest environment, with the highest pH (5.64) and relatively low concentrations of aggressive ions.

### 2.3. Weight-Loss Tests and Corrosion Morphology Analysis

The average corrosion rate of L415 steel was determined by weight-loss tests after 14 days of immersion in the test soils (with water content adjusted to 20 wt.%). After immersion, the specimens were removed, and the corrosion products were chemically cleaned according to the ASTM G1-03 standard [23]. Specifically, the corroded specimens were immersed in a cleaning solution (500 mL HCl + 500 mL distilled water + 3.5 g hexamethylenetetramine) for 10 min at room temperature. After cleaning, the specimens were rinsed with distilled water and ethanol, dried, and reweighed using an analytical balance with an accuracy of 0.1 mg to determine the final mass (m_1_).

The average corrosion rate (*CR*, in mm/year) was calculated using the following formula:

(1) CR=K×W/(A×T×D)

where *W* is the mass loss in grams (g), *K* is a constant (8.76 × 10^4^) for converting the corrosion rate to mm/year, *A* is the total exposed surface area of the specimen in cm^2^, *T* is the exposure time in hours (h), and *D* is the density of L415 steel, taken as 7.86 g/cm^3^.

After the removal of corrosion products, the corrosion morphology of the steel substrate was observed using a scanning electron microscope (SEM, TESCAN MIRA LMS, TESCAN ORSAY HOLDING, Brno, Czech Republic), with a focus on localized corrosion features such as pitting and ulcer-like corrosion.

### 2.4. Analysis of Corrosion Products

After the corrosion tests, specimens were gently cleaned with compressed air to remove loose soil particles. The surface and cross-sectional morphologies of the corrosion product films were examined by SEM. To identify the phase composition of the corrosion products, a portion of the product was scraped from the specimen surface for X-ray diffraction (XRD) analysis. The XRD measurements were performed using Cu Kα radiation at a tube voltage of 40 kV and a current of 40 mA, with a 2θ scan range from 10° to 90°.

### 2.5. Electrochemical Measurements

All electrochemical tests were conducted in simulated soil solutions using a CH760E electrochemical workstation (Shanghai Chenhua, Shanghai, China) in a conventional three-electrode cell. The epoxy-mounted steel specimen served as the working electrode (WE, with an exposed area of 1.0 cm^2^), a platinum sheet was used as the counter electrode (CE), and a saturated calomel electrode (SCE) was used as the reference electrode (RE). All potentials reported are related to the SCE.

Before each measurement, WE was immersed in the test solution for 1 h to allow the open circuit potential (OCP) to stabilize (e.g., potential fluctuation less than 2 mV within 10 min). Potentiodynamic polarization curves were scanned from −250 mV to +250 mV versus OCP at a scan rate of 0.5 mV/s. Electrochemical impedance spectroscopy (EIS) measurements were performed at the OCP, with a frequency range from 100 kHz to 10 mHz and an AC voltage perturbation of 10 mV. The EIS data were fitted and analyzed using ZsimpWin software 3.60. All experiments were conducted at room temperature and repeated at least three times to ensure reproducibility.

### 2.6. Slow Strain Rate Tensile (SSRT) Tests

The SCC susceptibility of L415 steel was evaluated by SSRT tests conducted in the four simulated soil solutions and in air (as a control environment) at room temperature. The tests were performed using a tensile testing machine (SINOTEST, Changchun, China, MFDL-50) at a constant strain rate of 1 × 10^−6^ s^−1^. During the tests in solution, the gauge section of the specimen was fully immersed in a corrosion cell containing the test medium.

After fracture, the ultimate tensile strength (UTS), the percentage of elongation (*δ*) and the percentage of reduction in area (*ψ*) were measured from the stress–strain curves and the fracture surfaces, respectively. The SCC susceptibility was quantified using the loss indices of ultimate tensile strength (*I*_UTS_), elongation (*I*_δ_), and reduction in area (*I*_ψ_), calculated by the following equations:

(2) $$I_{UTS}=(1-\frac{{\mathrm{UTS}}_{s}}{{\mathrm{UTS}}_{a}})\times 100\%$$

(3) $$I_{\delta }=(1-\frac{\delta_{s}}{\delta_{a}})\times 100\%$$

(4) $$I_{\psi }=(1-\frac{\psi_{s}}{\psi_{a}})\times 100\%$$

where *δ*_s_ and *ψ*_s_ are the elongation and reduction in area measured in the simulated soil solution, and *δ*_a_ and *ψ*_a_ are the corresponding values measured in air. Following the SSRT tests, the fracture surfaces were cleaned, dried, and examined by SEM to analyze the fracture mode.

## 3. Results and Discussion

### 3.1. Corrosion Rate and Surface Morphology

**Figure 3:**
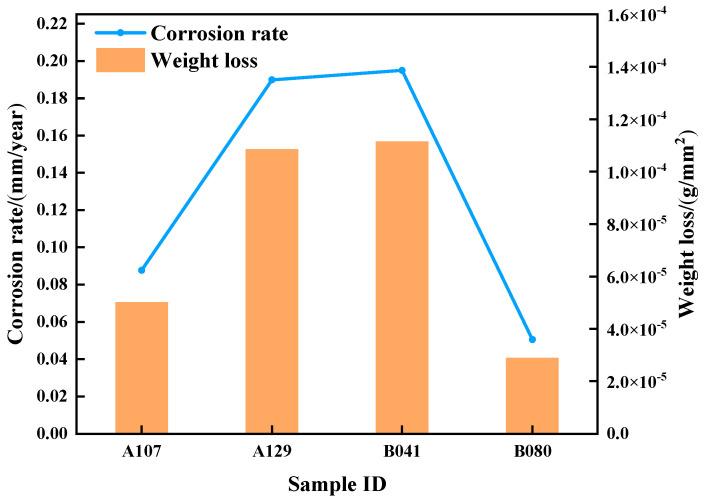
Average corrosion rates of L415 steel in the four different soil environments.

Figure 3 presents the average corrosion rates of L415 steel after 14 days of immersion, as determined by weight-loss measurements. The corrosion rate was found to be strongly dependent on the environment, following the order: B041 (0.1949 mm/a) > A129 (0.1898 mm/a) > A107 (0.0876 mm/a) > B080 (0.0505 mm/a). Notably, the corrosion rate in the B041 environment was nearly four times higher than that in the B080 environment.

These results indicate that the corrosivity of Gannan acidic soils is likely governed not by a single factor, but by the combined effects of pH, total ionic concentration, and specific aggressive anions (SO_4_^2−^, Cl^−^). The pronounced variation in corrosion rates among different soil samples implies that the protective properties and structural integrity of the corrosion product films formed under different environmental conditions vary substantially.

Based on these observations, it is hypothesized that in the most aggressive environments (B041 and A129), the extremely low pH and/or exceptionally high ionic concentration lead to the formation of loose, porous, and poorly protective rust layers. To verify this hypothesis, the morphology and composition of the corrosion products were systematically examined in the following section.

### 3.2. Characteristics and Protective Nature of Corrosion Product Films

**Figure 4:**
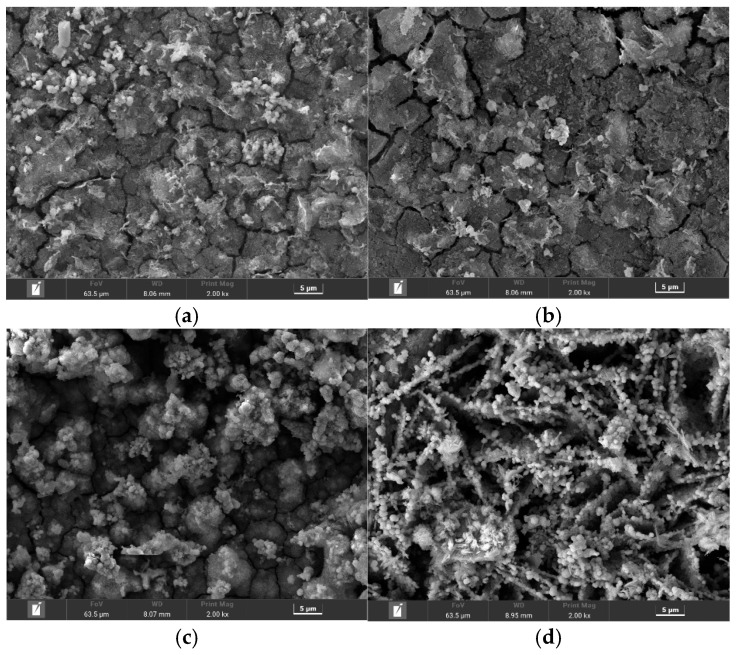
Surface morphology of corrosion product films formed on L415 steel in soils: (**a**) B041, (**b**) A129, (**c**) A107, and (**d**) B080.

To elucidate the mechanisms behind the widely divergent corrosion rates, the corrosion product films were systematically characterized. Figure 4 presents the surface SEM morphologies, revealing profound structural differences dictated by the environment. In the most aggressive solutions, A129 and B041, the films exhibited a complete loss of structural integrity. The film formed in the highly acidic A129 solution displayed a classic “mud-crack” morphology (Figure 4b), indicative of a brittle, dehydrated gel. The structural damage was even more severe in the high-sulfate B041 solution, which produced a fragmented and spalled layer with poor adhesion (Figure 4c). These extensive physical defects render the films non-protective, explaining the high corrosion rates observed.

In stark contrast, the films formed in the milder environments were macroscopically intact. The A107 solution, with its high chloride content, produced a film composed of porous, flake-like aggregates (Figure 4a). A distinct, highly crystalline morphology emerged in the least corrosive B080 solution, which consisted of an open, three-dimensional network of interwoven, needle-like crystals (Figure 4d), a typical structure for lepidocrocite (γ-FeOOH).

**Figure 5:**
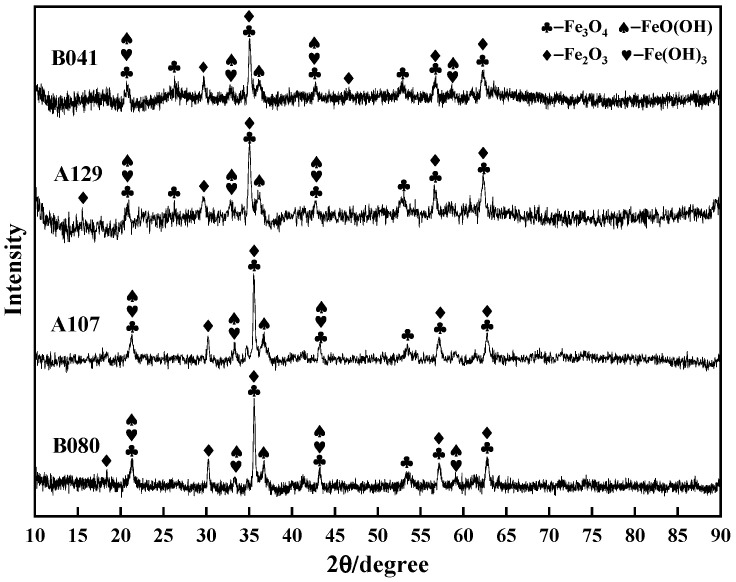
XRD patterns of corrosion products formed on L415 steel in the four different soil environments.

XRD analysis was performed to identify the phase composition of these morphologically distinct layers (Figure 5). The results confirmed that all films were primarily a mixture of lepidocrocite (γ-FeOOH), goethite (α-FeOOH), and magnetite (Fe_3_O_4_). However, the crystallinity varied dramatically and correlated directly with the observed morphologies. The XRD patterns for the severely cracked A129 and B041 films featured broad, diffuse peaks, indicating a substantial amorphous or poorly crystalline fraction. Conversely, the sharp and intense diffraction peaks from the B080 sample confirmed the high crystallinity of the well-defined γ-FeOOH needles observed in the SEM.

The protective quality of the rust layer, which is the decisive factor controlling the corrosion rate, is thus a function of both its physical integrity and its crystallinity. In the mild B080 environment, the higher pH and lower ionic concentration facilitated an ordered crystallization process, yielding a macroscopically continuous and well-crystallized γ-FeOOH film. Although microscopically porous, its superior structural integrity provided a more effective barrier against ion transport, resulting in the lowest corrosion rate. The A107 environment represents an intermediate case; the high Cl^−^ concentration is known to interfere with the formation of dense, protective layers, promoting the loose, less protective structure observed. This direct correlation between the environment, the film’s resulting structure and composition, and the final corrosion rate provides a clear mechanistic picture of the steel’s degradation process.

### 3.3. Electrochemical Corrosion Mechanism

**Figure 6:**
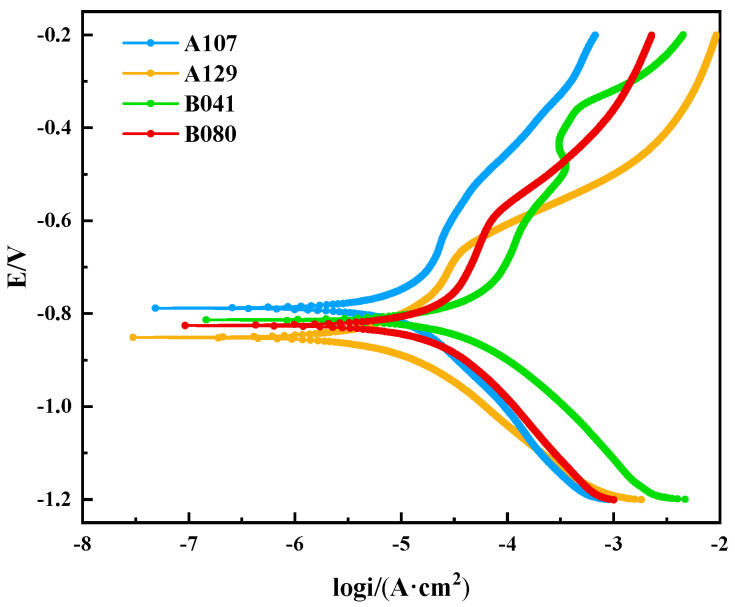
Polarization curves of L415 steel in the four different soil environments.

To quantitatively assess the corrosion behavior, electrochemical measurements were conducted. Figure 6 presents the potentiodynamic polarization curves after 14 days of immersion, with key parameters derived from Tafel extrapolation listed in Table 3. The corrosion current density (icorr), a direct indicator of corrosion rate, followed the order: B041 > A129 > A107 > B080, which aligns perfectly with the weight-loss results, providing mutual validation of the data. A notable finding is the performance of the B080 specimen. It exhibited relatively negative corrosion potential (Ecorr = −0.825 V) yet had the lowest corrosion rate. This apparent discrepancy highlights that the initial thermodynamic driving force is not the sole determinant of corrosion resistance. Instead, the superior performance of B080 is attributed to the formation of a particularly stable and protective surface film, which acts as a highly effective kinetic barrier that outweighs the strong thermodynamic tendency for corrosion.

**Figure 7:**
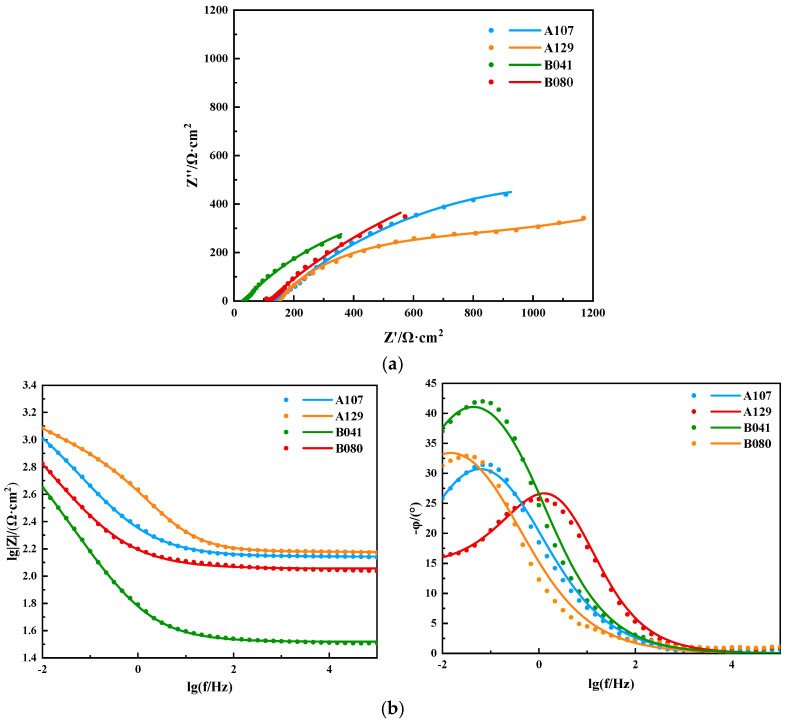
EIS spectra for L415 steel in the four simulated soil solutions: (**a**) Nyquist plots; (**b**) Bode plots.

Electrochemical impedance spectroscopy (EIS) was employed to further deconstruct the interfacial processes. The Nyquist plots (Figure 7a) feature a single, depressed capacitive semicircle for all specimens, characteristic of a charge-transfer-controlled corrosion process. The diameter of the semicircle, representing the total polarization resistance, varied significantly and followed the order: B080 >> A107 > A129 > B041. This trend, which is consistent with the Bode plots (Figure 7b), indicates that the B080 interface is substantially more resistant to corrosion than the others.

**Figure 8:**
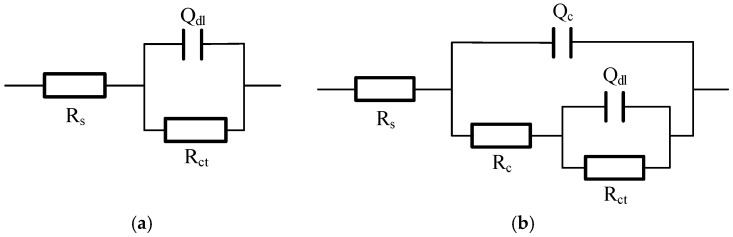
An equivalent electrical circuit model used for fitting the electrochemical impedance spectroscopy (EIS) data of L415 steel exposed in the four simulated soil solutions. (**a**) A107, B041, B080; (**b**) A129.

The EIS data were fitted using the equivalent electrical circuit (EEC) *R_s_*(*Q_c_R_c_*)(*Q_dl_R_ct_*), shown in Figure 8, where (*R_c_*, *Q_c_*) and (*R_ct_*, *Q_dl_*) represent the film and double-layer components, respectively. The fitted parameters, detailed in Table 4, provide a quantitative basis for the observed behaviors. The charge transfer resistance (*R_ct_*), which is inversely proportional to the corrosion rate, was inversely correlated with the *i*_corr_ values, once again confirming the kinetic trend. More importantly, the film resistance (*R_c_*) quantitatively reflects the film’s barrier properties. The B080 specimen yielded the highest R value, confirming it formed the most protective film. Conversely, the extremely low *R_c_* values for the A129 and B041 specimens are in excellent agreement with the defective “mud-cracked” and fragmented film structures observed via SEM, which offer negligible resistance to ion transport.

In summary, the corrosion of L415 steel in acidic soil environments proceeds via the anodic dissolution of iron (Fe ⟶ Fe^2+^ + 2e^−^) and the cathodic hydrogen evolution reaction (2H^+^ + 2e^−^ ⟶ H_2_). Among the environmental factors considered, pH exerts the primary influence on the corrosion rate, as it directly affects the charge transfer kinetics by increasing proton availability, as reflected in the variation of R_ct_.

Meanwhile, aggressive anions such as Cl^−^ and SO_4_^2−^ play a secondary but significant role by compromising the stability and protectiveness of the corrosion product films, thereby reducing R_c_. The observed differences in corrosion behavior across soil samples can therefore be attributed to the synergistic effect of low pH accelerating the corrosion reaction, and anionic species undermining the rust layer’s barrier function.

### 3.4. Stress Corrosion Cracking Susceptibility and Mechanism

**Figure 9:**
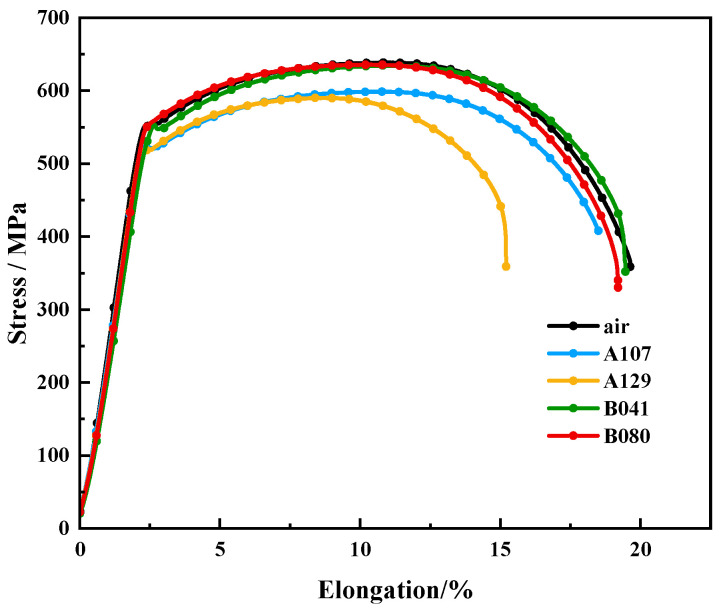
Stress–strain curves of L415 steel from SSRT tests in air and the various simulated soil solutions.

The susceptibility of L415 steel to environment-assisted cracking was evaluated using slow strain rate tensile (SSRT) tests. As shown by the stress–strain curves in Figure 9, all specimens tested in the simulated solutions exhibited a degradation in mechanical properties compared to the test in air, confirming a clear susceptibility to SCC. This degradation was most pronounced in the A129 solution, where the specimen experienced premature failure with a significant loss in both elongation and tensile strength.

**Figure 10:**
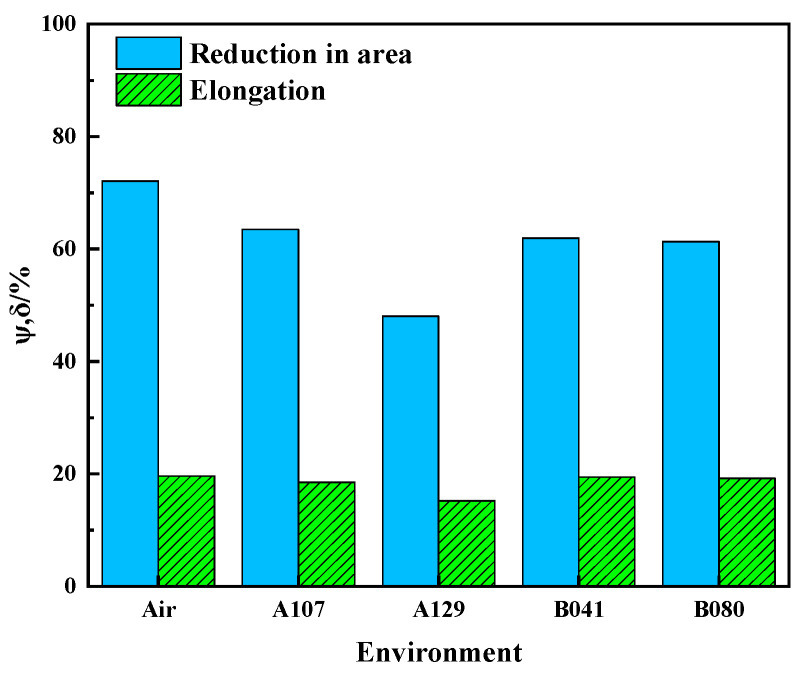
Comparison of the SCC susceptibility index (I_ψ_) of L415 steel in the various simulated soil solutions.

The SCC susceptibility was quantified using the indices for loss in elongation (I_δ_) and reduction in area (I_ψ_), summarized in Table 5 and Figure 10. The data reveals a strong environmental dependence, with the A129 solution inducing the most severe cracking (I = 33.30%). Based on the I_ψ_ index, the SCC susceptibility of L415 steel in the four environments follows the order: A129 > B080 > B041 > A107.

**Figure 11:**
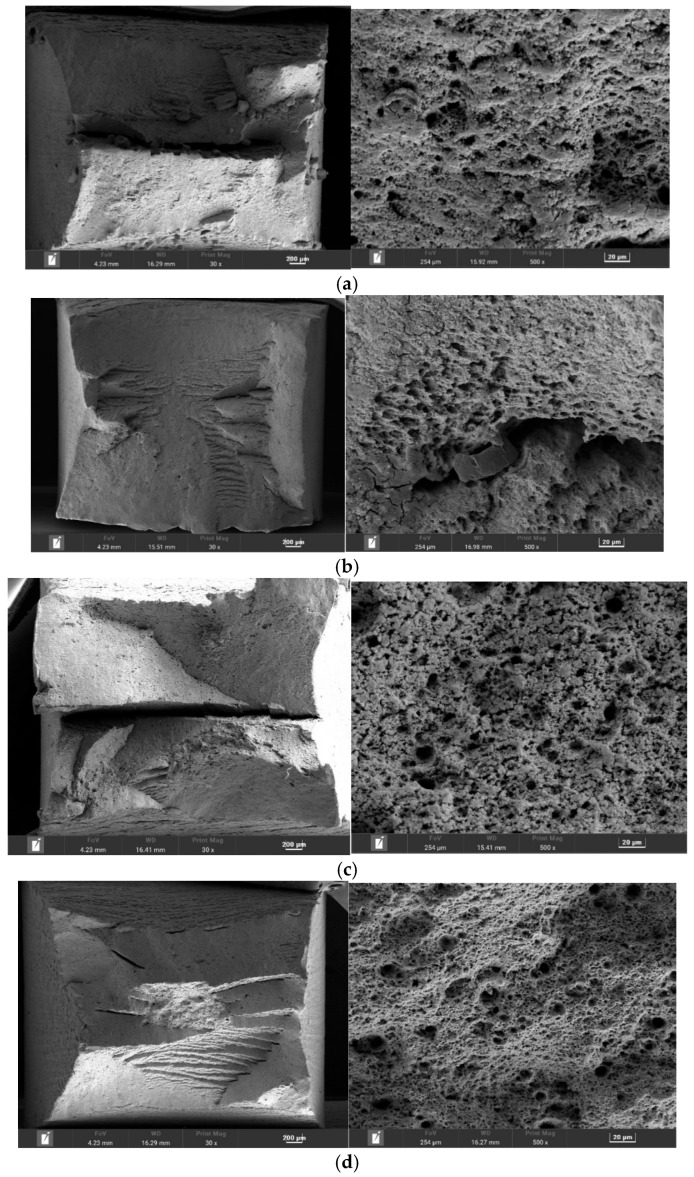
SEM micrographs showing the fracture surface morphologies of L415 steel after SSRT tests in: (**a**) A107, (**b**) A129, (**c**) B041, and (**d**) B080.

To elucidate the governing SCC mechanisms, the fracture surfaces were analyzed and correlated with the electrochemical conditions. The fractographic evidence (Figure 11) revealed distinct failure modes dependent on the environment. It can be observed that the microscopic fracture morphology is predominantly characterized by dimples in 4 solution environments, indicating that the fracture type of the L415 pipeline steel specimen is ductile fracture.

In the highly acidic A129 solution (pH 3.4), where susceptibility was highest, the dimples at the fracture site are shallower and sparser, with some areas exhibiting river patterns. The fracture surface was characterized by intergranular cracking (IGSCC). This morphology, coupled with the prolific cathodic hydrogen evolution kinetics observed in the polarization data for this environment, provides compelling evidence for hydrogen embrittlement (HE) as the dominant mechanism. In this scenario, the high concentration of H^+^ accelerates the hydrogen evolution reaction, increasing hydrogen atom uptake into the steel lattice. These atoms diffuse and accumulate at high-stress regions like grain boundaries, reducing cohesive strength and promoting brittle, intergranular failure.

In contrast, the fracture mode in the high-Cl^−^ A107 solution shifted to transgranular stress corrosion cracking (TGSCC), featuring quasi-cleavage facets and secondary cracks. This suggests a more complex mechanism involving the synergistic action of AD and HE. The high Cl^−^ concentration is known to locally disrupt the surface film, leading to pitting. Under tensile stress, these pits act as preferential crack initiation sites. The crack tip, a region of newly exposed and active metal, undergoes preferential dissolution (the AD component). Concurrently, the occluded chemistry within the crack can become more acidic, sustaining local hydrogen evolution and promoting hydrogen-assisted cracking at the crack tip (the HE component).

The specimens from the B041 and B080 environments exhibited intermediate susceptibility; the fracture surfaces were characterized by uniformly distributed, large and deep dimples, with only minimal evidence of brittle fracture features. This likely failed via a mixed-mode mechanism combining both HE and AD. The presence of brittle fracture features confirms the occurrence of environment-assisted cracking, with the relative contribution of HE versus AD likely varying depending on the specific interplay of pH and ionic composition in each environment.

## 4. Conclusions

This study demonstrated that the environmental chemistry of acidic soils is the governing factor that dictates the corrosion and SCC failure modes of L415 pipeline steel. The main conclusions are as follows:

1. The corrosion behavior of L415 steel was strongly influenced by environmental chemistry. In particular, the most severe corrosion rate (0.1949 mm/a) was recorded in acidic soils (pH < 4.5) with elevated SO_4_^2−^ levels, while the mildest corrosion (0.0505 mm/a) occurred in weakly acidic soils with low ionic content. This nearly four-fold variation confirms that a synergistic effect of low pH, high total ionic strength, and specific aggressive anions (e.g., SO_4_^2−^, Cl^−^) drives accelerated corrosion.
2. Electrochemical impedance spectroscopy (EIS) revealed that the corrosion kinetics were governed by two major factors: the resistance of the corrosion film (R_c_) and the charge transfer resistance (R_ct_). In aggressive environments, R_c_ and R_ct_ values were significantly reduced due to the formation of non-protective, porous films composed mainly of poorly crystalline γ-FeOOH and Fe_3_O_4_, as verified by XRD. In contrast, higher R_c_ and R_ct_ values were associated with dense, crystalline γ-FeOOH layers formed in less corrosive soils.
3. L415 steel exhibited signs of increased susceptibility to environment-assisted cracking under acidic conditions, as indicated by reduced ductility and brittle fracture features. The degree of susceptibility appeared to correlate with environmental acidity, suggesting that low pH plays a significant role in initiating damage.
4. The dominant cracking mechanism varied with the environment and reflects the combined effects of anodic dissolution (AD) and hydrogen embrittlement (HE). In strongly acidic, sulfate-rich soils, intergranular cracking features suggest that HE is the primary contributor, with H^+^ promoting hydrogen uptake. In high-chloride soils, the transition to transgranular stress corrosion cracking (TGSCC) indicates a synergistic mechanism of AD + HE, which is in line with the literature on SCC in carbon steels.

## Figures and Tables

**Table 1:** Chemical composition of L415 pipeline steel (wt.%).

	C	Si	P	S	Cr	Mn	Ni	Fe
L415	0.08	0.16	0.015	<0.003	—	1.61	—	Bal.

**Table 2:** Corrosion-Related Chemical Parameters of the Soil Samples.

Sample ID	pH	Cl^−^ (mg/L)	SO_4_^2−^ (mg/L)	HCO_3_^−^ (mg/L)	TDS (mg/L)
A129	3.4	6.45	55.92	/	144.73
B041	4.38	6.37	244.57	12.48	506.18
A107	4.9	29.66	3.19	12.95	69.03
B080	5.64	8.67	11.58	15.1	90.6

**Table 3:** Electrochemical parameters obtained from Tafel plots for L415 steel.

Solution ID	*E*_corr_ (V vs. SCE)	*i*_corr_ (A·cm^−2^)
A107	−0.788	1.66 × 10^−5^
A129	−0.832	2.76 × 10^−5^
B041	−0.814	4.07 × 10^−5^
B080	−0.825	7.96 × 10^−6^

**Table 4:** Fitted EIS parameters for L415 steel in the four simulated soil solutions.

Solution ID	*R_s_*/(Ω·cm^2^)	*Q_c_*/(F·cm^−2^)	*R_c_*/(Ω·cm^2^)	*Q_dl_*/(F·cm^−2^)	*R_ct_*/(Ω·cm^2^)	*R_p_*/(Ω·cm^2^)
A107	139.3	/	/	3.29 × 10^−3^	2025	/
A129	144	6.34 × 10^−4^	353.5	2.22 × 10^−3^	892.5	1246
B041	32.99	/	/	9.75 × 10^−3^	1029	/
B080	113.7	/	/	6.57 × 10^−2^	2700	/

**Table 5:** Mechanical properties and SCC susceptibility indices of L415 steel from SSRT tests.

Environment	UTS (MPa)	Time to Fracture (h)	Reduction in Area *ψ* (%)	Elongation *δ* (%)	I_UTS_ (%)	I_δ_ (%)	I_ψ_ (%)
Air (control)	638	72.6	72.09	19.6	0	0	0
A107	598	68.58	63.49	18.52	6.27	5.53	11.93
A129	591	56.27	48.08	15.22	7.37	22.35	33.3
B041	634	72.05	61.97	19.46	0.63	0.75	14.03
B080	635	71.12	61.33	19.21	0.47	2.03	14.92

## Data Availability

The data presented in this study are available on request from the corresponding author due to legal restrictions. The data are part of an ongoing study/are proprietary to our industry partner and are subject to confidentiality agreements.
